# TCRbeta1/TCRbeta2 (TRBC1/TRBC2) antibody pair for determining T-cell monotypia as a surrogate for clonality in formalin-fixed paraffin-embedded material

**DOI:** 10.1136/jcp-2026-210684

**Published:** 2026-04-30

**Authors:** Anuradha Kaistha, Jinlong J Situ, Shelley Evans, Margaret Ashton-Key, Graham Ogg, Elizabeth Soilleux

**Affiliations:** 1 Department of Pathology, University of Cambridge, Cambridge, UK; 2 Department of Cellular Pathology, Southampton University Hospitals NHS Trust, Southampton, UK; 3 MRC Weatherall Institute of Molecular Medicine, University of Oxford, Oxford, UK

**Keywords:** MONOCLONAL ANTIBODY, LYMPHOCYTES, LYMPHOMA, IMMUNOHISTOCHEMISTRY, DIAGNOSIS

## Abstract

**Aim:**

T-cell lymphomas are often histologically indistinguishable from benign T-cell infiltrates. Clonality testing is frequently required for diagnosis. It lacks the spatial context and is slow and expensive, relying on complex multiplexed PCR reactions, interpreted by scientists or pathologists with specialist molecular training. We set out to make monoclonal antibodies to develop a novel immunohistochemical test for T-cell lymphoma, analogous to the kappa/lambda assay for B-cell and plasma cell neoplasms.

**Methods:**

We developed a pair of highly specific monoclonal antibodies against the two alternatively used but very similar T-cell receptor β constant regions, TCRβ1 and TCRβ2 (encoded by the TRBC1 and TRBC2 gene segments). We demonstrate the feasibility of immunohistochemical detection of TCRβ1 and TCRβ2 in formalin-fixed, paraffin-embedded tissue as a novel diagnostic strategy for T-cell lymphomas.

**Results:**

Single immunostaining results are presented for 13 T-cell lymphomas and 8 benign T-cell populations, together with illustrative examples of TCRβ1/2 double immunostaining. Finally, we show that this single immunostaining is amenable to automated cell counting, permitting accurate calculation of the TCRβ2:TCRβ1 ratio.

**Conclusion:**

This novel assay can be used in a similar way to the kappa/lambda assay for B-cell and plasma cell neoplasms.

**WHAT IS ALREADY KNOWN ON THIS TOPIC**

- T-cell lymphomas often require complex, multiplex PCR clonality tests for definitive diagnosis. We know that each alpha-beta T cell expresses either a TCRbeta1 or TCRbeta2 constant region as part of its T-cell receptor, encoded by the *TRBC1* and *TRBC2* gene segments, respectively.

**WHAT THIS STUDY ADDS**

- This study validates a rabbit monoclonal anti-TCRbeta1, rabbit monoclonal anti-TCRbeta2 and chimeric mouse monoclonal anti-TCRbeta2 antibody in formalin fixed paraffin embedded sections of T-cell lymphomas and benign T-cell populations.

**HOW THIS STUDY MIGHT AFFECT RESEARCH, PRACTICE OR POLICY**

- This work has the potential to make the diagnosis of T-cell lymphoma cheaper and quicker, as well as allowing morphological and immunophenotypic features to be assessed alongside our surrogate for T-cell clonality, in a manner analogous to the widely used kappa/lambda assays for B-cell and plasma cell proliferations.

## Introduction

 T-cell lymphomas account for 10%–15% of lymphomas and frequently have a rapid and aggressive course.^1^ They are difficult, time-consuming and expensive to diagnose, so patients often require multiple biopsies, with months or years-long diagnostic delays impacting survival (reviewed in Soilleux *et al*).^1^

T-cell lymphomas composed of small-to-medium-sized cells (eg, peripheral T-cell lymphoma, angioimmunoblastic lymphoma and mycosis fungoides)^2^ are particularly difficult to distinguish, both morphologically and immunophenotypically, from benign lymphocytic infiltrates. Diagnosis often necessitates multiplexed PCR T-cell clonality analysis of DNA extracted from tissue, a complex test interpreted by specially trained staff members. Currently, clonality studies are only undertaken in large specialist centres and can delay diagnosis by several weeks. Compared with immunostained tissue on a slide, DNA-based testing precludes concomitant assessment of tissue architecture, morphology and immunophenotype of any cells suspected to be a clonal population.^1 3^

While T-cell lymphomas often require clonality studies for diagnosis, suspected B-cell lymphomas or plasma cell neoplasms can be assessed, by immunohistochemistry, in situ hybridisation or flow cytometry, for kappa and lambda expression.^4^,^6^ This phenomenon of light-chain monotypia (restriction to one light-chain type) acts as a surrogate for clonality and means that a much smaller proportion of suspected B-cell lymphomas and plasma cell neoplasms undergo clonality testing.

Around 95% of T cells bear an αβ T-cell receptor (TCR).^2^ Gene segment rearrangement at the TCRβ (*TRB*) locus produces T cells with either a TCRβ1 or TCRβ2 constant region, encoded by *TRBC1* or *TRBC2,* respectively.^7^,^9^ Immunohistochemical detection of the TCRβ1/2 proteins, in a manner analogous to kappa and lambda light chain detection in B cells, allows timely and cost-effective assessment of T-cell monotypia (TCRβ restriction), as a surrogate for T-cell monoclonality.^1 3^ In lymph nodes, around two-thirds of benign, mature T cells are CD4+CD8-, while almost all of the remainder are CD4-CD8+.^10^ T-cell lymphomas are often CD4+, less commonly CD8+, and occasionally CD4-CD8-.^2^ Assay sensitivity for TCRβ restriction in suspected malignant T-cell populations might be further increased by assessing TCRβ1/2 expression in individual T-cell populations separated on the basis of their CD4 and CD8 expression.

We previously published details of an antibody pair amenable to formalin-fixed paraffin-embedded (FFPE) immunostaining, suitable for assessing T-cell monotypia.^1^ Here we validate an improved rabbit monoclonal anti-TCRβ1/2 pairing, with a better performing anti-TCRβ2. We demonstrate their utility for single and double immunostaining, including with a chimeric mouse anti-TCRβ2 antibody. Finally, we show that this staining is amenable to automated cell counting, permitting accurate calculation of the ratio of TCRβ2-expressing to TCRβ1-expressing cells in each sample. We can then estimate the normal TCRβ2: TCRβ1 ratio range, and, as with the kappa/lambda assay, assume that T-cell populations with ratios outside this range are more likely to be malignant.

## Materials and methods

### Production of recombinant rabbit and mouse antibodies

As in a previous study,^1^ rabbits were immunised with TCRβ1- or TCRβ2-derived peptides to produce TCRβ1-specific (ROX7) and TCRβ2-specific (ROX13) rabbit IgG antibodies. Recombinant antibodies were expressed in HEK293T cells and purified by size-exclusion HPLC (Superdex 200, GE28-9909-44, Sigma Aldrich, Gillingham, UK) to achieve >98% purity. Chimeric mouse antibody (MOX13) was produced by recombinant switching of the C-regions of the ROX13 antibody.

### Cell culture, western blotting and the immunostaining of immortal T-cell and B-cell lines

Jurkat (TCRβ1-expressing), CEM, MOLT-4, HPB-ALL (TCRβ2-expressing)^11^ and Daudi (B-cell; TCRβ1/β2-negative) immortalised lymphoid cell lines were used for Western blot analysis.^1^ Briefly, cells were washed with ice-cold PBS and lysed in RIPA buffer supplemented with a protease inhibitor cocktail (cOmplete Mini, EDTA-free; Roche; 11836170001; 100×stock). Protein concentrations were determined using the BCA assay (Pierce BCA Protein Assay Kit; Thermo Fisher Scientific, A65453). Equal amounts of protein were resolved by SDS–PAGE and transferred onto 0.45 µm PVDF membranes (Invitrogen LC2005). Membranes were blocked for 1 hour at room temperature in 5% fat-free dry milk in TBS containing 0.1% Tween-20 (TBST) and incubated overnight at 4°C with rabbit anti-TCRβ2 (ROX13; 0.001 µg/µL), mouse anti-TCRβ2 (MOX13; 0.001 µg/µL) or rabbit anti-β-tubulin (CST 2128T; 1:1000). After TBST washes, membranes were incubated for 1 hour at room temperature with HRP-conjugated anti-rabbit or anti-mouse secondary antibodies (Jackson ImmunoResearch). Signals were developed using SuperSignal West Atto Ultimate Sensitivity substrate (Thermo Fisher Scientific) and imaged with the ChemiDoc Touch Imaging System (Bio-Rad).

A pellet of each of the above cell lines was produced by centrifugation, clotted with human plasma (National Health Service Blood Transfusion Service, Cambridge, UK) and bovine thrombin (50 U/mL; BTUB291, Diagnostic Reagents, Thame, UK), marked in a specific colour with ‘Cancer Diagnostics Tissue Marking Dye’ (CellPath, Newtown, Powys, UK) and processed to paraffin.^1^ 3 μm paraffin sections were immunostained on charged microscope slides.

### FFPE clinical tissue samples

Histological patient samples (table 1) were obtained from the Cambridge University Hospitals NHS Foundation Trust Human Research Tissue Bank or ‘Research Histology’ in the Department of Cellular Pathology, University Hospital Southampton NHS Foundation Trust, Southampton, UK, with full ethical approval (IRAS: 162057; PI: Professor E. Soilleux).^1^

**Table 1:** Analysis of TCRβ2+:TCRβ1+cell immunostaining ratios in tissue sections containing benign and malignant T-cell populations (shaded cells and italicised text, respectively), performed using the QuPath and StarDist software,^12 13^ with human oversight

Case number	Site	Diagnosis	Type of case	Field 1	Field 2	Field 3	Average IHC cell TCRβ2:TCRβ1 (x:1)
1	Tonsil	Benign, reactive	Benign	0.99	1.14	1.35	1.14
2	Lymph node	Benign, reactive	Benign	0.82	1.23	0.69	0.86
3	Lymph node	Benign, reactive	Benign	0.78	0.96	1.91	1.06
4	Lymph node	Benign, reactive	Benign	0.48	1.15	1.15	0.78
5	Skin (temple)	Lichenoid keratosis	Benign (inflammatory)	0.75	0.30	0.68	0.49
6	Buccal mucosa	Lichen planus	Benign (inflammatory)	1.08	1.06	1.07	1.07
7	Lymph node	T-cell/histiocyte-rich large B-cell lymphoma	Benign (tumour-infiltrating)	0.76	1.51	1.39	1.11
8	Lymph node	Classic Hodgkin lymphoma	Benign (tumour-infiltrating)	1.19	1.23	1.42	1.25
*9*	*Lymph node*	*Peripheral T-cell lymphoma, NOS*	*T-cell lymphoma*	*0.06*	*0.14*	*0.08*	*0.09*
*10*	*Lymph node*	*Peripheral T-cell lymphoma, NOS*	*T-cell lymphoma*	*34.17*	*20.93*	*9.87*	*16.82*
*11*	*Lymph node*	*Peripheral T-cell lymphoma, NOS*	*T-cell lymphoma*	*8.06*	*6.15*	*10.11*	*7.78*
*12*	*Skin (ear*)	*Indolent CD8+T cell lymphoma*	*T-cell lymphoma*	*67.17*	*11.50*	*5.00*	*9.94*
*13*	*Skin (forehead*)	*CD4+cutaneous T-cell lymphoma, unclassifiable*	*T-cell lymphoma*	*0.48*	*0.27*	*0.27*	*0.31*
*14*	*Skin (back*)	*Sézary syndrome*	*T-cell lymphoma*	*3.14*	*11.37*	*4.36*	*4.72*
*15*	*Skin (elbow*)	*Primary cutaneous anaplastic large cell lymphoma or transformed mycosis fungoides*	*T-cell lymphoma*	*10.56*	*3.05*	*8.64*	*5.58*
*16*	*Skin (scrotum*)	*Transformed mycosis fungoides*	*T-cell lymphoma*	*0.02*	*0.04*	*0.02*	*0.02*
*17*	*Skin (arm*)	*Transformed mycosis fungoides*	*T-cell lymphoma*	*10.82*	*18.65*	*12.22*	*13.17*
*18*	*Skin (back*)	*Mycosis fungoides*	*T-cell lymphoma*	*1.96*	*3.10*	*2.46*	*2.42*
*19*	*Vulva*	*CD30+cutaneous T cell lymphoma, unclassifiable*	*T-cell lymphoma*	*0.11*	*0.09*	*0.01*	*0.02*
*20*	*Skin (back*)	*Cutaneous T cell lymphoma*	*T-cell lymphoma*	*7.06*	*5.67*	*2.57*	*4.24*
*21*	*Skin (buttock*)	*CD8 positive cutaneous T cell lymphoma, unclassifiable*	*T-cell lymphoma*	*8.46*	*46.17*	*21.80*	*16.15*

As benign T-cell populations showed a small excess of TCRβ2+ compared with TCRβ1+ cells, TCRβ2:TCRβ1 (rather than TCRβ1:TCRβ2) ratios were quantified. We have previously published quantitative real-time reverse transcription PCR and in situ hybridisation, determining the *TRBC2:TRBC1* ratios in these samples,^1^ all of which corroborate the immunostaining results in this table. All counting was performed on sections immunostained singly for either TCRβ1 or TCRβ2.

IHC, immunohistochemistry.

### Immunostaining

Chromogenic immunohistochemical staining of cell pellets and patient samples was performed on a Leica Bond RX or Roche Ventana Discovery automated immunostainer, using rabbit IgG monoclonal antibodies against TCRβ1 (ROX7, 0.4 µg/mL) and TCRβ2 (ROX13, 2 µg/mL) or mouse IgG monoclonal antibodies against TCRβ2 (MOX13, 2 µg/mL). Antibodies were incubated for 60 min following heat-induced epitope retrieval for 60 min in alkaline buffer (Epitope Retrieval Solution 2; Cat. No. AR9640, Leica Biosystems, Newcastle-upon-Tyne, UK) or CC2 (ROX7) or CC1 (ROX13) (Cat. No. 950-224/950-223, Roche Diagnostics, Burgess Hill, UK). Detection was performed using the Polymer Refine Detection System (Cat. No. DS9800, Leica Biosystems, Newcastle-upon-Tyne, UK) or Optiview Detection System (Cat. No. 760-099, Roche Diagnostics, Burgess Hill, UK).

For double immunohistochemical staining, undertaken as proof-of-concept only, sequential staining was performed using antibodies ROX7, ROX13 or MOX13 as above, detected with the Polymer Refine Detection System (brown diaminobenzidine (DAB) chromogen), followed by staining with a second primary mouse or rabbit antibody (ROX13, MOX13, anti-CD4 (clone 4B12) or anti-CD8 (clone 4B11) (Leica, Newcastle, UK)), detected with the Bond Intense R Detection System (Cat. No. DS9263, Leica Biosystems, Newcastle-upon-Tyne, UK) (red chromogen). For two cases of cutaneous T-cell lymphoma (cases 19 and 21, table 1), the following double immunostains were performed: ROX7/ROX13, ROX7/CD4, ROX7/CD8, ROX13/CD4, ROX13/CD8. ROX7/ROX13 and ROX7/MOX13 immunostaining was performed on two cases of benign tonsillar tissue.

Images were acquired at 100× and 400× magnification using an Infinity 2 camera (Lumenera, Ottawa, ON, Canada) mounted on a BX53 microscope (Olympus, Tokyo, Japan).

### Analysis of histological results

Three non-overlapping fields of serial sections immunostained with anti-TCRβ1 and anti-TCRβ2 antibodies were photographed at 400×, and the photographs used for quantitative analysis of immunostaining. Automated cell counting was performed in QuPath, using a pretrained StarDist model, under human supervision.^12 13^ For each field, pixel size was adjusted to 0.4×0.4 µm and stain vectors were estimated from a representative region. Cells were classified based on mean DAB immunostaining intensity (strong/weak) and size (medium-to-large (>50 μm^2^)/small (≤50 μm^2^)), these size categories having been determined from haematopathologist (ES)-annotated images of tissue sections. Positive cells of all sizes and intensities were counted. Counts were manually validated by three observers (blinded to diagnosis), including a practising haematopathologist (ES).

## Results

### Western blotting and immunostaining of FFPE pellets of immortalised cell lines

**Figure 1:**
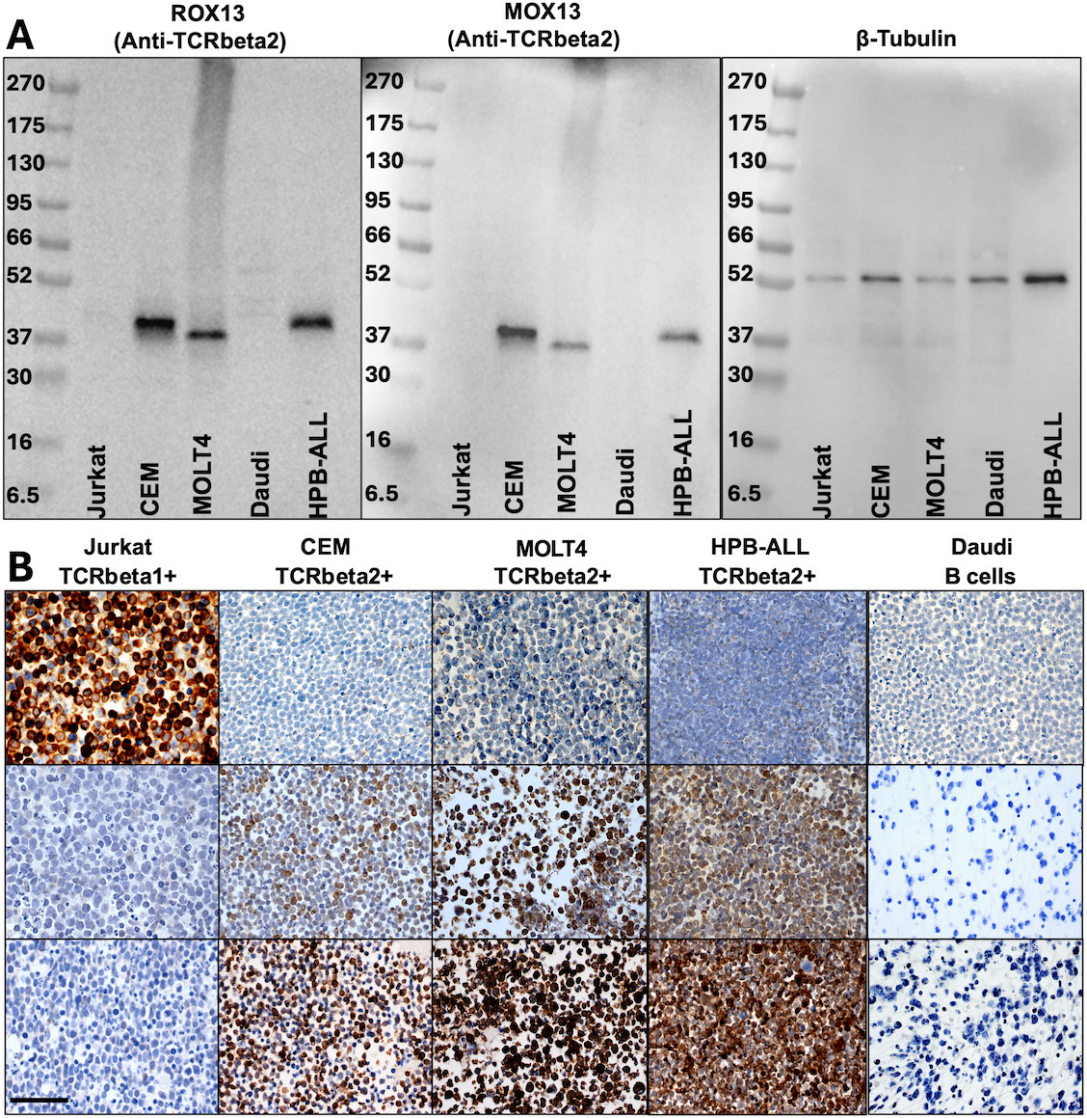
Validation of TCRβ isotype-specific monoclonal antibodies by Western blotting and immunohistochemistry. (**A**) Western blot demonstrating the specificity of TCRβ2-specific monoclonal antibodies ROX13 (rabbit) and MOX13 (mouse). Antibodies were validated using Jurkat (TCRβ1^+^), CEM (TCRβ2^+^), MOLT4 (TCRβ2^+^), Daudi B (TCRβ-negative) and HPB-ALL (TCRβ2^+^) cell lines. Monoclonal rabbit anti-β-tubulin is used as a protein loading control. (**B**) Chromogenic immunostaining of FFPE cell pellets from Jurkat, CEM, MOLT4, Daudi and HPB-ALL cell lines. The high specificity of ROX7 (TCRβ1-specific), ROX13 (TCRβ2-specific) and MOX13 (TCRβ2-specific) antibodies is demonstrated. Target antigens are visualised as brown staining using diaminobenzidine (DAB), with nuclei counterstained blue by haematoxylin. Scale bar (bottom left) represents 50 μm and pertains to all images in B. Compared with our previously published ROX11 anti-TCRβ2 monoclonal antibody,^1^ the ROX13 and MOX13 antibodies give stronger positive immunostaining, with minimal non-specific staining, at a much lower concentration than ROX11. FFPE, formalin-fixed paraffin-embedded.

Western blotting and immunostaining of FFPE pellets of immortalised cell lines demonstrated the TCRβ2 specificity of our OX13 monoclonal antibodies, in both the original rabbit (ROX13) and chimeric mouse (MOX13) form (figure 1). These anti-TCRβ2 antibodies gave strong and highly specific immunostaining at a much lower concentration than our previously published ROX11 anti-TCRβ2 antibody.^1^ We confirmed the previously demonstrated TCRβ1-specificity of the rabbit monoclonal antibody, ROX7.^1^

### Utility of the anti-TCRβ1/anti-TCRβ2 pair in separating benign and malignant (lymphomatous) T-cell populations

**Figure 2:**
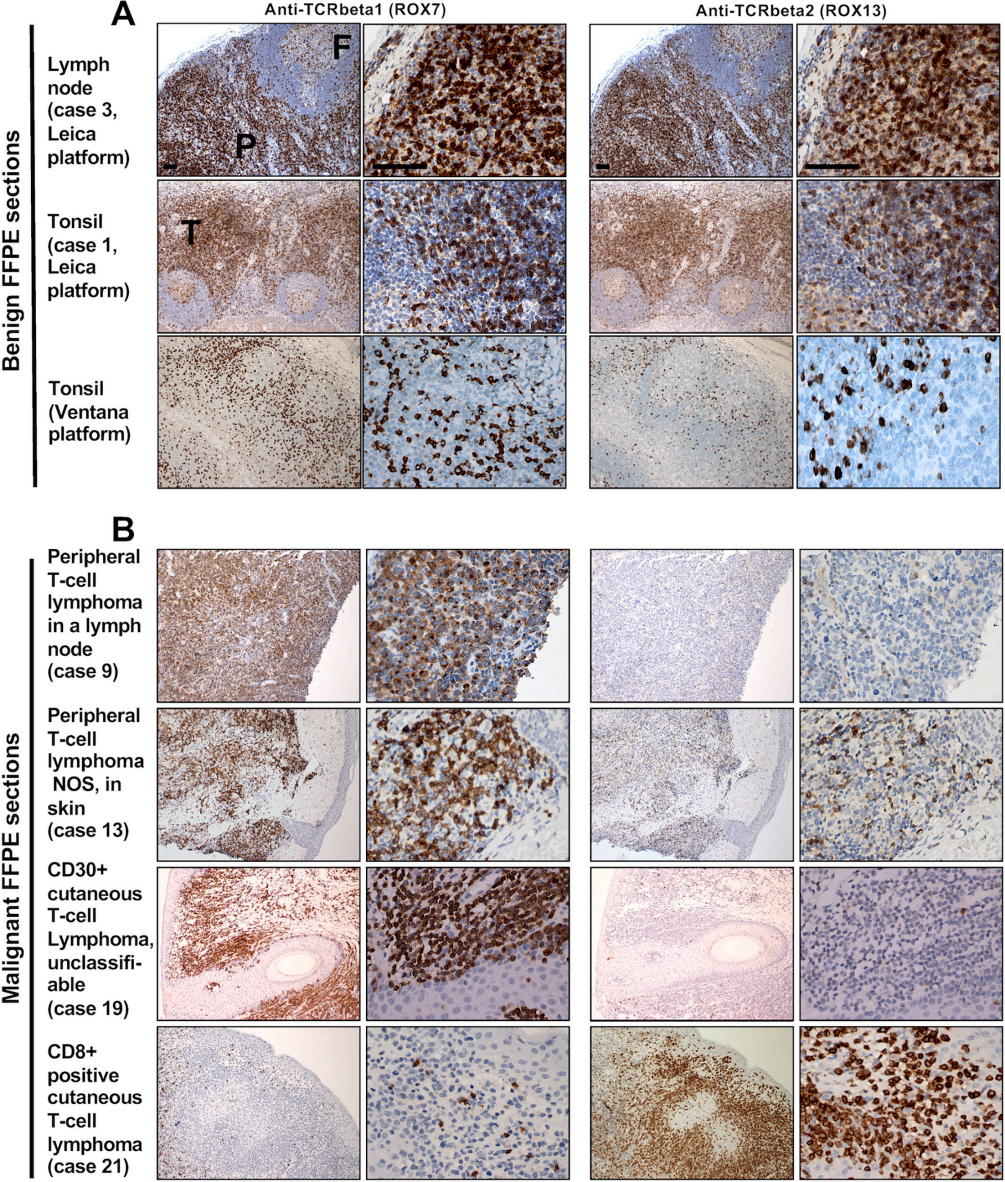
Validation of anti-TCRβ1/2 monoclonal antibodies for immunohistochemistry on FFPE samples. Representative images of benign (**A**) and malignant (**B**) tissue sections immunostained with ROX7 (TCRβ1 specific; left panels) and ROX13 (TCRβ2 specific; right panels). Benign sections show positive immunostaining (brown) of T cells in the lymph node paracortex (P), tonsillar T zone (T) and among the scattered T-cell population in B-cell follicles (example labelled F). Comparable numbers of cells are stained positively with each antibody, on both the Leica (top two rows) and Ventana immunostaining platforms (lower row). In (B), the upper three panels represent TCRβ1-restricted lymphomas, whereas the bottom panel represents a TCRβ2-restricted lymphoma. Compared with clonality testing, our TCRβ1/2 immunostaining has the advantage of allowing assessment of the distribution (lesional architecture) of TCRβ1/2+T cell infiltrates. Furthermore, TCRβ-restriction can be assessed in cell populations with specific morphology. Case 9, for example, shows that the large lymphoid cell population is TCRβ1-restricted. Case numbers refer to the cases described in table 1. Scale bars in top row panels represent 50 μm and pertain to all panels in the same column. FFPE, formalin-fixed paraffin-embedded.

**Figure 3:**
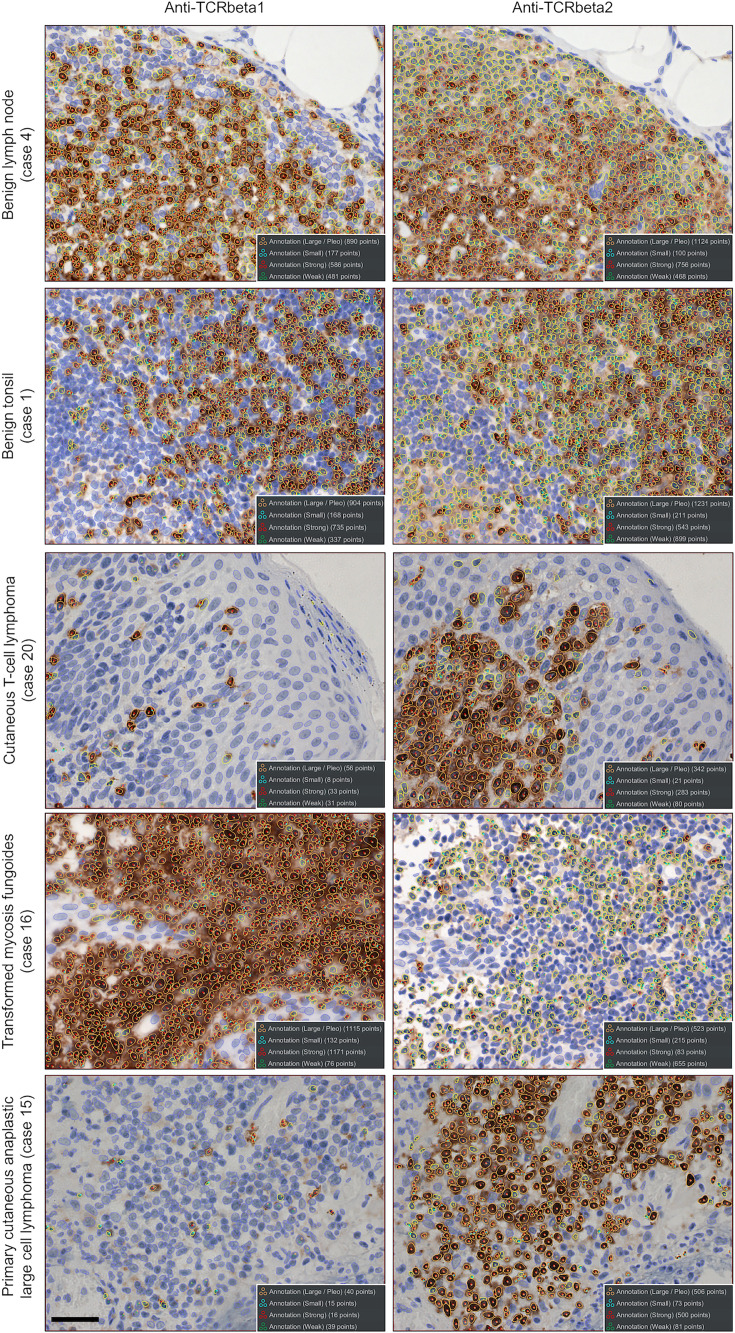
Analysis of tissue sections containing benign and lymphomatous T-cell populations using an automated cell counting workflow. Tissue sections were immunostained (brown) with anti-TCRβ1 (ROX7) and anti-TCRβ2 (ROX13) antibodies with a blue haematoxylin nuclear counterstain. Cell detection was performed using StarDist. Cells were categorised based on staining intensity and size, then quantified. Annotated outlines around cells indicate diaminobenzidine (DAB) immunostaining intensity: orange (strongly stained), yellow (weakly stained) and blue (negative). Dots overlying the nuclei indicate exactly which nuclei were counted, and their size and DAB immunostaining intensity (key in bottom right of each panel). The corresponding case number of each image and diagnosis is indicated to the left of each row. Scale bar in bottom left-hand panel represents 50 μm and pertains to all panels. Case 20, a cutaneous T-cell lymphoma with morphological features of mycosis fungoides, demonstrates clear TCRβ2-restriction of its epidermotropic T-cell population. This underlines the advantage of our immunohistochemical surrogate for clonality testing over conventional DNA-based clonality analysis,^3 7^ because our immunohistochemical approach permits simultaneous assessment of cellular morphology and immunophenotype.^1^

We undertook single ROX7 and ROX13 immunostaining of 8 benign (4 reactive lymphoid tissue; 2 inflammatory and 2 tumour-infiltrating T-cell populations) and 13 lymphoma samples (table 1), selected because more tissue was available from these than from a core or punch biopsy, permitting RNA extraction for quantitative PCR in parallel with immunostaining.^1^ In the benign T-cell populations in FFPE lymph node and tonsil tissue shown in figure 2A, there are roughly equal numbers of TCRβ1+ (ROX7+) and TCRβ2+ (ROX13+) T cells, both in the paracortex of lymph nodes, the T-zones of tonsils, and among the follicular helper T cells of B-cell follicles. Examples of T-cell lymphomas with clear TCRβ1 or TCRβ2 restriction are shown in figure 2B. Compared with clonality testing, our immunostaining has the advantages of allowing assessment of both the cellular morphology and distribution (lesional architecture) of TCRβ1/2+T cell infiltrates. Examples of this can be seen in case 9 (figure 2) which shows TCRβ1-restriction of the large lymphoid cells, while case 20 (figure 3) demonstrates clear TCRβ2-restriction of its epidermotropic T-cell population.

### Application of an automated cell counting method (with human oversight) to the immunostained sections

While TCRβ restriction is obvious in many T-cell lymphomas, the admixture of benign T cells, which constitute a roughly equal TCRβ1+/2+mixture, may confound this assessment. In this situation, counting of cells may be necessary and can prove labourious. We therefore tested whether publicly available, user-friendly software, which is free to download and use, could perform this task on our TCRβ1/2 single-stained sections. A combination of the QuPath and StarDist software, with human oversight, was used to quantify positively immunostained cells in three paired (TCRβ1/2) serial section images (figure 3).^12 13^ As benign T-cell populations showed a small excess of TCRβ2+compared with TCRβ1+cells, TCRβ2:TCRβ1 (rather than TCRβ1:TCRβ2) ratios were quantified. The mean TCRβ2:TCRβ1 ratios in benign T-cell populations were between 0.49:1 and 1.25:1, with the ratios in all lymphoma samples falling outside this (table 1). We thus demonstrated that an automated cell counting method (with human oversight) could be applied to TCRβ1/2-immunostained FFPE sections.

### Anti-TCRβ1/2 double immunostaining could facilitate diagnosis

**Figure 4:**
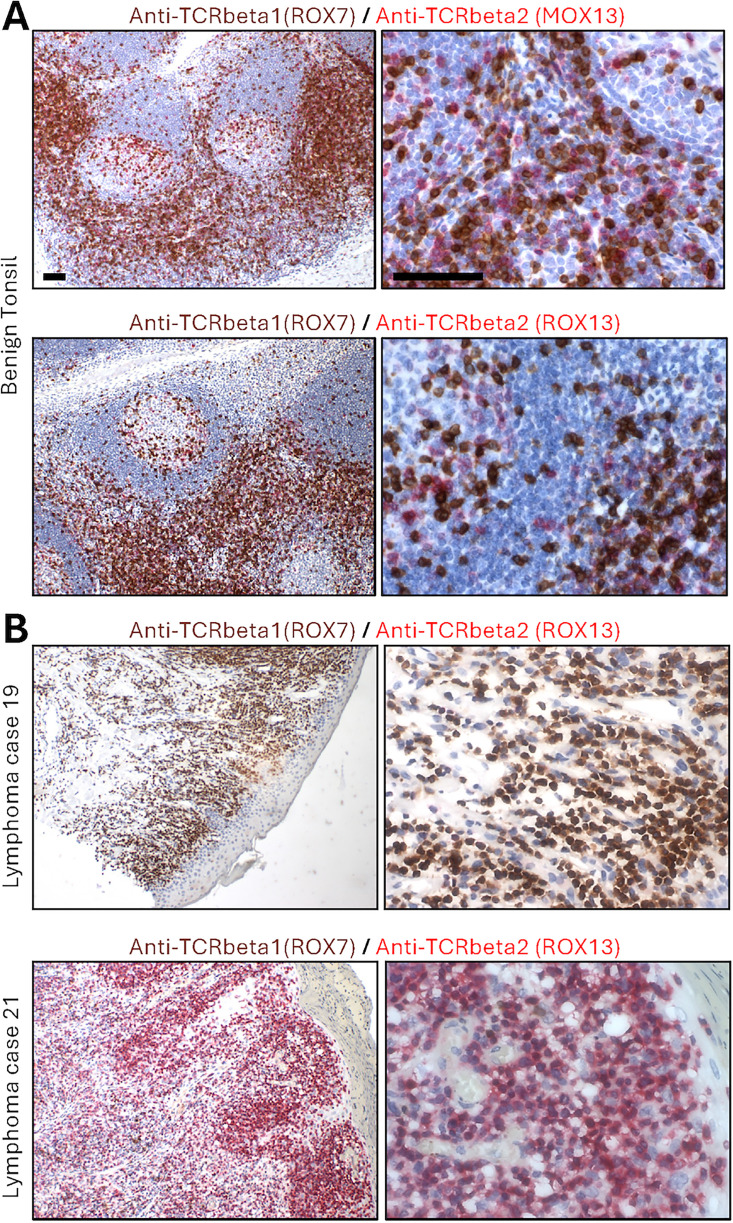
Validation of anti-TCRβ1/2 monoclonal antibodies for double immunostaining with rabbit anti-TCRβ1 (brown) and mouse/rabbit anti-TCRβ2 (red) antibodies, on benign (**A**) and malignant (**B**) T-cell populations. (**A**) Tonsil (upper panel) shows roughly equal numbers of TCRβ1+ (brown) and TCRβ2+ (red, detected with mouse antibody). (**B**) TCRβ1-restricted CD30+ cutaneous T-cell lymphoma, unclassifiable (case 19; upper panel) and TCRβ2-restricted cutaneous lymphoma (case 21; lower panel) immunostained. Panels in (B) show immunostaining with rabbit anti-TCRβ1 (brown) and mouse anti-TCRβ2 (red) antibodies. Scale bars in top row panels represent 50 μm and pertain to all panels in the same column. Single TCRβ1 and TCRβ2 immunostaining of cases 19 and 21 is shown in figure 2B, permitting comparison with the double immunostaining.

The chimeric mouse version of our anti-TCRβ2 antibody (MOX13) gave very similar staining to that of the rabbit parent antibody (ROX13), so we sought to test the potential utility of both these antibodies in double immunostaining with our anti-TCRβ1 (ROX7) antibody on the Leica Bond Rx platform on two cases of benign tonsillar tissue. As proof of concept, figure 4A shows very similar results with the two antibody pairs, with similar numbers of TCRβ1+ and TCRβ2+ cells. Double immunostaining of T-cell lymphomas with the rabbit anti-TCRβ1/2 (ROX7/ROX13) antibody pair permitted visualisation of TCRβ restriction in two examples of cutaneous T-cell lymphoma (cases 19 and 21 (details in table 1; figure 4B). Single TCRβ1 and TCRβ2 immunostaining of cases 19 and 21 is shown in figure 2B, permitting comparison with the double immunostaining.

### Potential utility of anti-TCRβ1/2 double immunostaining with CD4 or CD8

**Figure 5:**
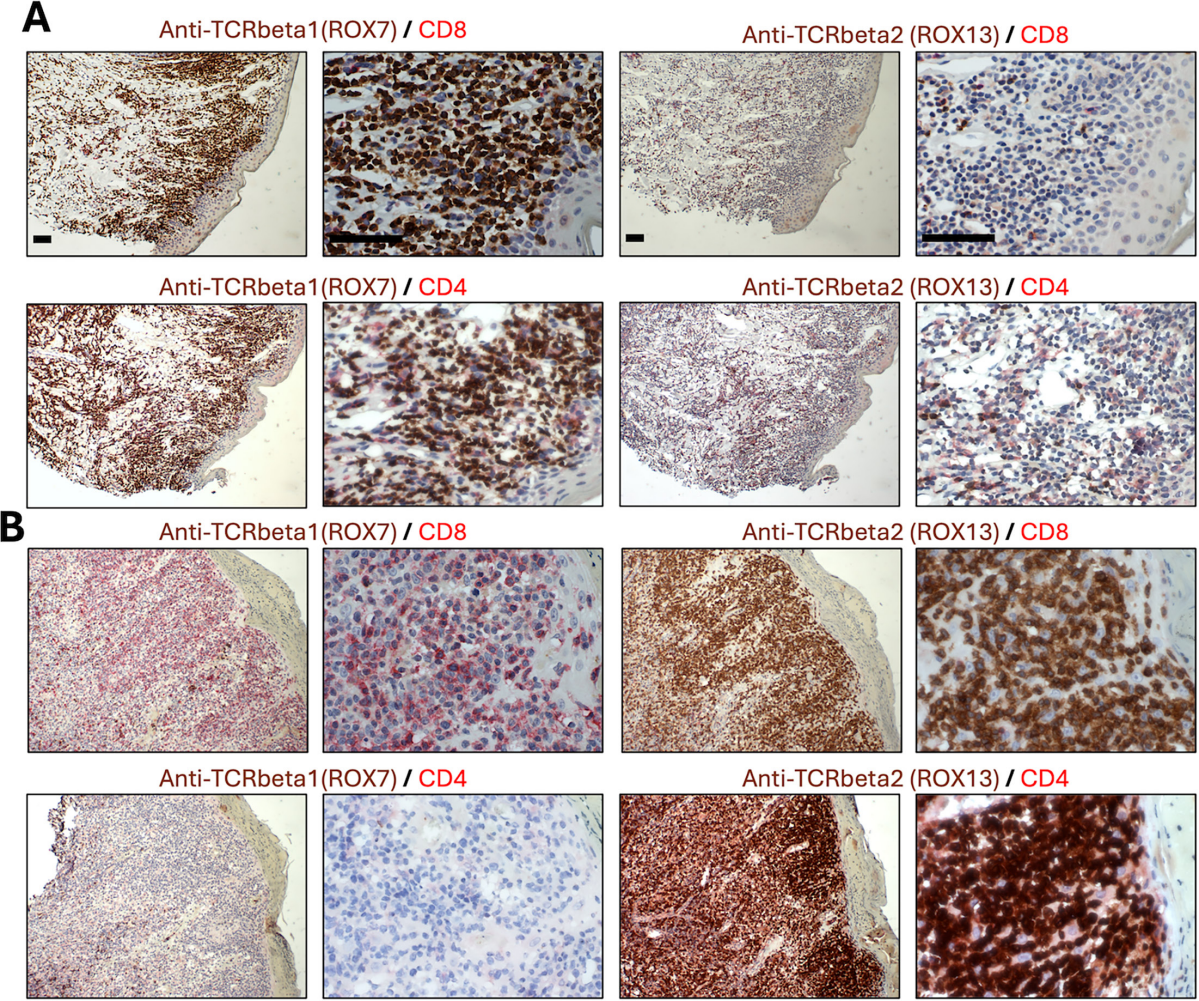
Validation of anti-TCRβ1/2 monoclonal antibodies for distinguishing tumour-infiltrating from malignant T-cell populations. Double immunostaining of cutaneous lymphomas showing TCRβ1-restriction (case 19; panels in A) or TCRβ2-restriction (case 21; panels in B), performed using anti-TCRβ1 (ROX7; brown) or anti-TCRβ2 (ROX13; brown) antibodies in combination with anti-CD8 (upper panels; red) or anti-CD4 (lower panels; red) antibodies. Immunostaining demonstrates that the TCRβ1-restricted lymphoma cells are CD4+, whereas the TCRβ2-restricted lymphoma cells are CD8+. Scale bars in top row panels represent 50 μm and pertain to all panels in the same column. Single TCRβ1 and TCRβ2 immunostaining of cases 19 and 21 is shown in figure 2B, permitting comparison with the double immunostaining.

As proof of concept, we show two examples of TCRβ1/2 double immunostaining, together with anti-CD4/CD8 (cases 19 and 21; details in table 1). Figure 5 shows these two cutaneous T-cell lymphomas, one weakly CD4+, TCRβ1-restricted (case 19; figure 5A) and one CD8+, TCRβ2-restricted (case 21; figure 5B).

## Discussion

Here, we validate a pair of antibodies for single and double immunostaining that could substantially improve and expedite the diagnosis of T-cell lymphoma, which, for example in our institution, might decrease time-to-diagnosis by around 2 weeks. The diagnosis of T-cell lymphoma relies initially on immunostaining multiple serial sections with immunophenotypic markers, such as CD2, CD3, CD4, CD5, CD7, CD8, CD25 and/or CD30, among others. Adding our antibodies to the diagnostic armoury requires just two additional serial sections, although double or multiplexed immunostaining with our antibodies and CD4 or CD8 could also be undertaken, if, in future, it is shown to be clinically useful. Immunostaining with our antibodies, unlike clonality testing, can be undertaken in centres without a specialist haematopathology service. While some guidelines (eg, in the UK) mandate only diagnosing lymphomas in specialist haemato-oncology diagnostic units,^14^ the antibodies could be very valuable in non-specialist centres, to triage specimens for referral to specialist centres.

TCRβ-restriction was visually obvious without counting cells in many of our lymphoma cases, but, in some lymphomas, a significant tumour-infiltrating benign T-cell population confounds analysis. We show that such cases are amenable to automated cell counting. This approach permitted calculation of a benign/malignant TCRβ2:TCRβ1 ratio cut-off of 0.49–1.25:1. This ratio will be further refined with a subsequent larger study of T-cell populations in various non-lymphomatous pathologies, across a range of body sites and patient ages and through double immunostaining. Subsequent studies will also need to focus on a broad range of types of T-cell lymphoma, including diagnoses with very mixed T-cell populations, such as angioimmunoblastic T-cell lymphoma and small samples, such as punch and core biopsies. These studies should be undertaken with and without the use of automated counting.

Larger studies involving multiple pathologists are also required to determine the utility of TCRβ1/TCRβ2 double immunostaining and double staining combinations of TCRβ1 and TCRβ2 with CD4 and CD8, particularly because co-localisation of the chromogens may make interpretation difficult. While the TCRβ1 and TCRβ2 rabbit antibodies can be used together with appropriate detection systems, our chimeric mouse anti-TCRβ2 antibody, which performs very similarly to the parent rabbit anti-TCRβ2 antibody, can facilitate double immunostaining in situations in which antibodies of two different species are required.

Although one TCRβ1/2 antibody pair has been used in flow cytometry,^15^ this and our previously published antibody pairing are the only examples, to our knowledge, of an anti-TCRβ1/anti-TCRβ2-specific monoclonal antibody pair that works on FFPE material, making it suitable for solid haematopathological specimens. This approach, like kappa and lambda detection for B-cell lymphoma and myeloma, will expedite and improve the triage and diagnosis of T-cell lymphomas, particularly cutaneous lymphomas and those with small/medium cellular morphology.

## Data Availability

All data relevant to the study are included in the article or uploaded as supplementary information.

## References

[1] Soilleux EJ, Rodgers DT, Situ JJ (2024). Demonstration of T-Cell Monotypia Using Anti-TCRbeta1/2 (*TRBC1*/2) Immunostaining as a Rapid and Cost-Effective Alternative to PCR-Based Clonality Studies for the Diagnosis of T-Cell Lymphoma. Diagnostics (Basel).

[2] WHO Classification of Tumours Editorial Board (2024). WHO classification of tumours: haematolymphoid tumours part B. 5th edition.

[3] Davies K, Staniforth J, Xie WH (2020). Advances in the assessment of T-cell clonality. Diagn Histopathol (Oxf).

[4] Minca EC, Wang H, Wang Z (2015). Detection of immunoglobulin light-chain restriction in cutaneous B-cell lymphomas by ultrasensitive bright-field mRNA in situ hybridization. J Cutan Pathol.

[5] McNicol AM, Farquharson MA, Lee FD (1998). Comparison of in situ hybridisation and polymerase chain reaction in the diagnosis of B cell lymphoma. J Clin Pathol.

[6] Levy R, Warnke R, Dorfman RF (1977). The monoclonality of human B-cell lymphomas. J Exp Med.

[7] van Dongen JJM, Langerak AW, Brüggemann M (2003). Design and standardization of PCR primers and protocols for detection of clonal immunoglobulin and T-cell receptor gene recombinations in suspect lymphoproliferations: report of the BIOMED-2 Concerted Action BMH4-CT98-3936. Leukemia.

[8] Laydon DJ, Bangham CRM, Asquith B (2015). Estimating T-cell repertoire diversity: limitations of classical estimators and a new approach. *Philos Trans R Soc Lond B Biol Sci*.

[9] Lefranc M-P, Lefranc Gr (2001). ScienceDirect (Online Service). The T cell receptor factsbook. Factsbook series.

[10] McBride JA, Striker R (2017). Imbalance in the game of T cells: What can the CD4/CD8 T-cell ratio tell us about HIV and health?. PLoS Pathog.

[11] Leiden JM, Dialynas DP, Duby AD (1986). Rearrangement and expression of T-cell antigen receptor genes in human T-lymphocyte tumor lines and normal human T-cell clones: evidence for allelic exclusion of Ti beta gene expression and preferential use of a J beta 2 gene segment. Mol Cell Biol.

[12] Bankhead P, Loughrey MB, Fernández JA (2017). QuPath: Open source software for digital pathology image analysis. Sci Rep.

[13] Schmidt U, Weigert M, Broaddus C (2018). Cell Detection with Star-Convex Polygons. Lect Notes Comput Sc.

[14] Snowden JA, O’Connell S, Hawkins J (2017). Haematological cancers: improving outcomes. A summary of updated NICE service guidance in relation to Specialist Integrated Haematological Malignancy Diagnostic Services (SIHMDS). J Clin Pathol.

[15] Horna P, Weybright MJ, Ferrari M (2024). Dual T-cell constant β chain (TRBC)1 and TRBC2 staining for the identification of T-cell neoplasms by flow cytometry. Blood Cancer J.

